# The Effect of Hydroxyapatite Inclusion on the Chemical, Physical and Biological Properties of Polyhydroxybutyrate/Chitosan Scaffolds

**DOI:** 10.3390/polym18091073

**Published:** 2026-04-29

**Authors:** Yulia Zhuikova, Vsevolod Zhuikov, Dolgor Khaydapova, Balzhima Shagdarova, Valery Varlamov

**Affiliations:** 1 Research Center of Biotechnology of the Russian Academy of Sciences, Moscow 119071, Russia; vsevolod1905@yandex.ru (V.Z.); shagdarova.bal@gmail.com (B.S.); varlamov@biengi.ac.ru (V.V.); 2 Faculty of Soil Science, M.V. Lomonosov Moscow State University, Moscow 119991, Russia; dkhaydapova@yandex.ru

**Keywords:** polyhydroxybutyrate, chitosan, hydroxyapatite, scaffolds, rheology, AFM, WAC, biodegradation, biocompatibility

## Abstract

This study focuses on the development and comprehensive evaluation of the physicochemical, mechanical, and biological properties of composites based on polyhydroxybutyrate (PHB), chitosan (Ch), and hydroxyapatite (HA) for biomedical applications. DSC and FTIR spectroscopy showed that the addition of hydroxyapatite did not significantly affect the structure of the materials, but AFM data revealed a change in the surface morphology. Variations in RMS roughness ranging from 13 to 150 nm were observed for chitosan and the composites. The density of the HA-containing samples was 0.06–0.067 g/cm^3^, which is higher than that of the unfilled composite (0.056 g/cm^3^). Optimal hydrophilic properties (contact angle 38.9°) and elasticity (damping factor 0.064) were recorded for the sample with 10% HA (PChHA10). The water absorption varied: the addition of chitosan increased the value to 7.5 g/g, compared to 2.7 g/g for pure PHB, while HA slowed the swelling kinetics (more than 180 min). A biodegradation study revealed that samples containing 10–20% HA exhibited the highest stability in an enzymatic environment, while further increases in HA content resulted in increased degradation rates. The PChHA10 is considered to offer the balanced combination of properties. The potential applications of this material in medicine include its use as a scaffold for the *in vitro* cultivation of osteoblasts and chondrocytes, as well as for implantation in models of bone and cartilage defects *in vivo*.

## 1. Introduction

Current trends in the development of new biomaterials require new approaches to creating scaffolds for tissue engineering that can effectively mimic the properties of the extracellular matrix and promote the regeneration of damaged tissues [1,2]. Therefore, the development of materials based on natural polymers, their blends, and composites remains an extremely relevant area of research [3,4]. Despite the widespread use of synthetic polymers, their application is associated with a number of limitations, including long degradation times, the potential toxicity of degradation products, and insufficient biocompatibility [5,6]. These factors are driving the search for alternatives among naturally occurring compounds, important examples of which are chitosan (Ch) and polyhydroxybutyrate (PHB), which possess a unique set of biofunctional properties [7].

Polyhydroxybutyrate is a non-toxic, biocompatible, and biodegradable polyhydroxyalkanoate (PHA) used in biomedical fields such as wound healing, the development of controlled-release drug delivery systems, and tissue engineering [8,9]. Previously, we proposed a method for producing 3D-materials from PHB using the TIPS method. It was shown that this preparation method, compared to traditional leaching, leads to modifications in certain structural and physical properties of PHB gels and scaffolds [10]. However, the high degree of crystallinity, hydrophobicity, and relatively long biodegradation time of PHB still significantly limit its use in its native form [11].

Chitosan (Ch) is a linear polysaccharide consisting of alternating glucosamine and N-acetyl-D-glucosamine units linked by β-(1-4) glycosidic bonds. Chitosan exhibits hydrophilicity, rapid biodegradation, good biocompatibility, mucoadhesion, as well as antimicrobial, fungicidal, sorption, and hemostatic properties [12]. The polycationic nature of chitosan enables its ability to interact electrostatically with negatively charged components of cell membranes, which makes it a promising material for biomedical applications [13,14]. Despite the relative ease of producing chitosan-based scaffolds, its use in an unmodified form is limited due to poor mechanical properties and excessive liquid absorption capacity for a number of biomedical applications [15]. A promising approach to resolving this problem involves combining polymers in composite materials, which allows for the mitigation of the limitations of individual components and, in some cases, the achievement of a synergistic effect [16]. Nevertheless, existing methods for producing PHB/Ch composites often require the use of toxic organic solvents, complex and expensive equipment, and a labor-intensive process of selecting optimal conditions, which underscores the need to develop more efficient and safer approaches to creating biocomposite scaffolds [17,18].

One way to further improve polymer composites is to incorporate components of a different nature, such as minerals, which can impart new properties to the materials. Hydroxyapatite (HA; Ca_10_(PO_4_)_6_(OH)_2_), being the main mineral component of bone tissue and consisting primarily of calcium and phosphate [19], serves as a physiologically compatible filler for bone-replacement materials. HA is characterized by a high surface-to-volume ratio, non-toxicity in certain quantities, osteointegration capacity, non-immunogenicity, biocompatibility, and bioactivity [20]. The incorporation of HA into chitosan-based composite matrices is widely practiced to enhance biocompatibility, improve mechanical properties, and increase osteoregenerative potential [21,22], and some studies have noted the presence of antibacterial activity in such composites [23]. At the same time, research is being conducted to create PHB/HA composites using various methods. For example, it has been [24] demonstrated that certain mechanical properties of the resulting composites exceeded those of the individual components.

The development of composites compatible with tissue engineering requirements is of particular relevance. We hypothesize that the addition of hydroxyapatite to the PHB/chitosan blend will help overcome the limitations of existing biopolymers by combining their properties with those of the inorganic component in composite materials. The addition of HA to PHB/chitosan-based composites can enhance their bioactivity and functionality; however, such systems have been previously studied only to a limited extent. There is a need for more comprehensive data on the effects of composition on physicochemical, rheological, and biological properties, as well as on degradation kinetics under conditions that mimic physiological ones. A key feature of this project is the use of acetic acid as an available and low-toxicity common solvent, which aligns with the global trend toward “green chemistry.” In addition, we do not require any special equipment to create the scaffolds. Thus, the aim of this study was to prepare a series of PHB/chitosan/hydroxyapatite composite mixtures and to investigate the effect of varying hydroxyapatite content on the physicochemical, rheological properties of the scaffolds, their structure and surface characteristics, cytocompatibility and their biodegradability under conditions mimicking physiological ones, which is important for predicting their subsequent practical application. The successful completion of this research will result in the creation of materials with optimal properties suitable for use in the regeneration of bone and cartilage tissue.

## 2. Materials and Methods

### 2.1. Materials

PHB (Biomer Co, Krailing, Germany) with a molecular weight (MW) of 317 kDa, crab chitosan with MW of 1000 kDa and a degree of deacetylation of 85% (Bioprogress LLC, Shchelkovo, Russia), and HA in the form of a nanopowder with a particle size of less than 200 nm (CAS No: 12167-74-7, Sigma-Aldrich, St. Louis, MO, USA) were used to create the scaffolds. Other reagents used in the study include PBS (Rosmedbio, St. Petersburg, Russia), glacial acetic acid (Ekos-1, Moscow, Russia), chloroform (Ekos-1, Moscow, Russia), sodium bicarbonate (molar mass 84.01 g/mol, Sigma-Aldrich, St. Louis, MO, USA), chicken lysozyme (CAS No: 12650-88-3 Sigma-Aldrich, St. Louis, MO, USA), and porcine pancreatic lipase (CAS No: 9001-62-1, Sigma-Aldrich, St. Louis, MO, USA).

### 2.2. Preparation of PHB/Ch/HA Scaffolds

Polymer scaffolds were prepared based on our proposed method [10], with modifications. PHB film, pre-precipitated in chloroform, was ground, and glacial acetic acid was added (1 mL of acid per 25 mg of PHB). Hydroxyapatite powder was submitted in various mass ratios (5, 10, 20, 50 wt% for samples PChHA5, PChHA10, PChHA20, PChHA50, respectively). The mixture was heated to 120 °C while stirring, during which the PHB fragments completely dissolved. After that, a 2% chitosan solution in 2% acetic acid was added dropwise until a PHB/Ch ratio of 4:1 was reached; the mixture was stirred vigorously for 2 min and was then poured into a glass Petri dish and cooled in air for 10 min. As a result, the composition transformed into a physical gel as the temperature decreased. To remove excess acid, the gel was repeatedly immersed in a 1% sodium bicarbonate solution. The gel was then freeze-drying. The resulting dry scaffolds were stored at 3 °C for further analysis.

The PCh scaffold, which was prepared using the method described above, but without the addition of HA; the PHB scaffold (without the addition of HA or Ch); and the chitosan (Ch) scaffold were prepared for use as control samples. It was prepared by pouring a 2% solution of chitosan in 2% acetic acid into a Petri dish, followed by freeze-drying.

### 2.3. FTIR Spectroscopy

The FTIR spectra were obtained using a Spectrum Two FTIR spectrometer (PerkinElmer, Waltham, MA, USA) in attenuated total internal reflection mode. A LiTaO_3_ IR detector and a standard optical system with a resolution of 0.5 cm^−1^ were used, along with KBr windows, to collect data in the spectral range of 4000–350 cm^−1^.

### 2.4. Differential Scanning Calorimetry

The thermophysical properties of the samples were analyzed using a DSC 204 F1 Phoenix calorimeter (NETZSCH-Gerätebau GmbH, Selb, Germany). The temperature range was 25–200 °C, and the scanning rate was 10 °C/min. An aluminum crucible was used. The analysis was performed in a nitrogen atmosphere at a flow rate of 40 mL/min.

### 2.5. Atomic Force Microscopy

The surface structure of the scaffolds was examined in air in semi-contact mode using atomic force microscopy on an NtegraPrima device (NT-MDT SI, Moscow, Russia). The samples were probed using NSG01 series cantilevers (TipsNano, Moscow, Russia) with a stiffness of 1.45–15.1 N/m and a resonance frequency ranging from 87 to 230 kHz. Images were acquired and analyzed using the Nova software 1.1.0.1910 (NT-MDT SI, Moscow, Russia). The root mean square surface roughness was calculated as the average value for at least 5 scans per sample; the scanning area was 10 μm × 10 μm.

### 2.6. The Contact Angle of Scaffolds and Films

The standard sessile drop method was used to determine the contact angle values. The samples included both fully dried polymer films and gel samples immediately after preparation (prior to freezing and drying). Drops of distilled water (10 μL, at least 5 drops per sample) were placed on the surface of the samples, then photographed and analyzed using the Fiji software 2.17.0 [25]. At least 3 replicates of each sample were used, and the results were presented as the mean value and standard deviation.

### 2.7. Water Absorption Capacity (WAC)

The dried scaffolds were weighed previously to obtain the dry weight of the scaffold (m_dry). Next, each sample was placed in distilled water, then removed, excess liquid was blotted with filter paper, and the sample was weighed (m_wet). Water Absorption Capacity (g/g) was calculated using the following equation:

WAC = (m_wet − m_dry)/m_dry,(1)

The result was presented as a change in water absorption over time (10, 20, 30, 60, 120, 180, 1440 min). At least 3 samples of each type were used for each measurement.

### 2.8. Porosity and Density of Scaffolds

Porosity and density were calculated as follows in [26]. Distilled water was used as the liquid to fill the pores. First, the weight of the scaffolds immersed in a specified volume of water was measured. To fill all pores with water, the scaffolds were deaerated using a series of short air-pumping cycles. These cycles were repeated until air bubbles ceased to form on the surface of the scaffolds. Porosity (ε, %) was calculated using the equation:

ε = (V1 − V3)/(V2 − V3) × 100%,(2)

Density (d, g/cm^3^) was calculated using the equation:

d = m_dry/(V2 − V3),(3)

where m_dry is the weight of the dry scaffold (g), V1 is the initial volume of water (mL), V2 is the total volume occupied by water and the scaffolds (mL), and V3 is the volume of water after the scaffolds were removed from the liquid (mL).

### 2.9. Rheological Behavior

The rheological properties were investigated using a PHYSICA MCR-302 rheometer (Anton Paar GmbH, Graz, Austria) at a constant temperature of 20 °C with a 25 mm diameter plate–plate geometry. Scaffold samples were prepared in the form of discs with a diameter of 25 mm and a height of 3 mm. The dried scaffolds were pre-soaked in a PBS for 30 min. The deformation amplitude was determined using an amplitude test and was set to 0.05%. A frequency test was conducted using a frequency sweep in the range of 0.1–100 rad/s with a fixed amplitude, and the values of the storage and loss moduli, and complex viscosity were determined as a function of angular frequency. The results were processed and presented using OriginPro 2018 SR1 b.9.5.1.195 software (OriginLab Corporation, Northampton, MA, USA).

The Young’s modulus under compression was calculated. A compressive force was applied to each test piece, and the amount of deformation was calculated. The results were converted into stress–strain values. The following relationships were used for this:

Relative compression = (Gap_0_ − Gap)/Gap_0_, (4)

where Gap_0_ and Gap are the thicknesses of the test pieces before and after compression (mm);

Normal pressure (Pa) = Normal force/(πr^2^), (5)

where Normal force is the compressive force (N), and r is the sample radius (0.0125 m). Young’s modulus was calculated from the slope of the Normal pressure–Relative compression curve. At least three measurements were taken for each sample type, and the results are presented as the average value.

### 2.10. Biodegradation of Scaffolds in PBS and Enzyme Solution

Dried and weighed scaffolds were incubated in PBS, as well as in a solution of lipase (0.25 mg/mL) and lysozyme (13 mg/L) in PBS [27]. The medium was replaced with fresh medium every three days. Sodium azide (2 g/L) was added to prevent microbial contamination. At the end of the incubation period, the samples were rinsed with distilled water, dried to constant weight, and weighed. Weight measurements were taken on days 1, 3, 7, 30, and 60. For each control point, three samples of each type were used.

### 2.11. Assessment of Cytocompatibility

Sterile scaffolds were placed in a 96-well plate and seeded with MSCs (3 × 10^3^ cells/well). For the Alamar Blue assay, a stock solution of resazurin (0.15 mg/mL) (Alfa Aesar, Shanghai, China) was prepared in HBSS (HyClone, Logan, UT, USA). To exclude the contribution of cells that had not attached to the samples but were growing on the bottom of the culture plate wells, the scaffolds were transferred to clean wells containing 90 μL of DMEM medium and 10 μL of stock resazurin solution prior to the start of the assay. The samples were incubated with agitation for 2 h in a CO_2_ incubator at 37 °C. After incubation, 90 μL of the stained solution was transferred from the wells to clean wells and the optical density was measured using a Hidex Chameleon plate spectrophotometer (LabLogic, Sheffield, UK). At an excitation wavelength of 540 nm, the fluorescence emission wavelength was set to 585 nm. To estimate the number of cells in the samples, a calibration curve was constructed showing the relationship between fluorescence intensity and the number of cells per well in duplicate. For calibration, cells were seeded into wells without samples in the following quantities: 500; 150; 3500; 7000; and 10,500 cells/well.

### 2.12. Statistical Analysis

Statistical analysis was performed using OriginPro 2018 SR1 b.9.5.1.195 (OriginLab Corporation, Northampton, MA, USA) using one-way analysis of variance (ANOVA) with Brown–Forsythe test (due to unequal variances and sample sizes), followed by Holm–Sidak post hoc correction for multiple comparisons and Student’s t-test at a significance level of *p* < 0.05. Data presented in tables and figures are shown as mean ± SD, unless otherwise specified.

## 3. Results

In this study, composite scaffolds consisting of chitosan polysaccharide and PHB polyester and containing varying amounts of hydroxyapatite powder were prepared. The effect of hydroxyapatite addition on the main physicochemical, structural, and biological properties of the polymer scaffold was evaluated.

### 3.1. FTIR

**Figure 1:**
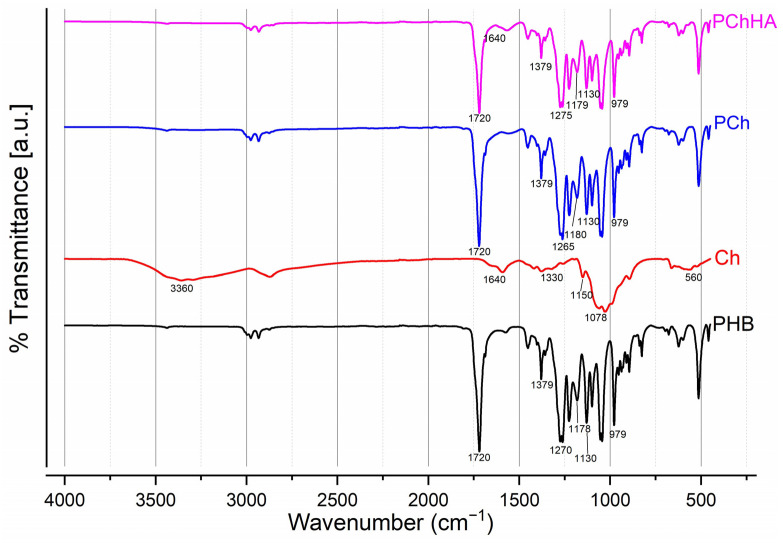
FTIR spectra for PHB, Ch, PCh, and PChHA10.

Figure 1 shows the spectra of four materials: PHB, Ch, and the PCh and PChHA combinations. By comparing them, differences and similarities can be observed.

The PHB spectrum exhibited a sharp peak in the region of 1720 cm^−1^. This corresponds to the valence vibrations of the carbonyl group (C=O) of the ester. A peak is also visible in the region of 1270 cm^−1^ (C-O-C bonds). The series of peaks at 1178 cm^−1^ and 1130 cm^−1^ can be attributed to C–O and C–C bonds. The observed peaks at 979 and 1720 cm^−1^ are often attributed to bonds characteristic of the polymer’s crystalline component, while the peak at 1178 cm^−1^ corresponds to vibrations of groups in the amorphous component [28]. Chitosan exhibited a broad absorption band in the 3200–3500 cm^−1^ region with a peak at 3360 cm^−1^. This corresponds to valence vibrations of hydroxyl groups (O–H) and amino groups (N–H). The peak in the region of 1640 cm^−1^ is characteristic of Amide I (C=O vibrations). A broad band with a peak at 1078 cm^−1^ was also observed, which is characteristic of C-O-C (1150–1160 cm^−1^) and C-O (1078 cm^−1^) bonds. The spectrum for the PCh blend was an overlap of the polymer spectra. A spectrum characteristic of PHB is clearly visible in the region of 1720 cm^−1^. In addition, a slight shift toward the low-frequency region was observed for this spectrum, which is presumably evidence of hydrogen bonding between the PHB carbonyl and the hydroxyl/amino groups of chitosan. The amide region of chitosan (1550–1650 cm^−1^) also decreased, as NH_2_ groups could participate in the formation of hydrogen bonds with PHB. In the 3600–3200 region, there was a slight increase in peak intensity, which is due to the influence of chitosan hydroxyl groups (O–H) and amino groups (N–H). Additionally, relative to PHB, slight peak shifts were observed, which can be explained by the influence of chitosan.

**Figure 2:**
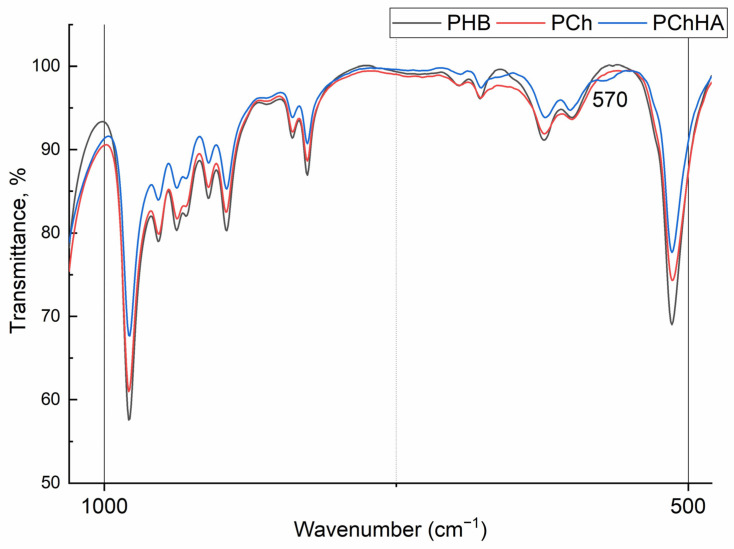
FTIR spectra for PHB, PCh, and PChHA10 at a wavenumber of 400–1000 cm^−1^.

Finally, the spectrum of the PChHA10 resembles that of PCh. It also exhibits a band at 1640 cm^−1^ characteristic of chitosan (amide I), and an intense peak at 1720 cm^−1^ characteristic of PHB. The doublets of hydroxyapatite are located in the 600–560 cm^−1^ region, but they are of low intensity and barely visible in the spectrum (Figure 2).

### 3.2. DSC Analysis

**Figure 3:**
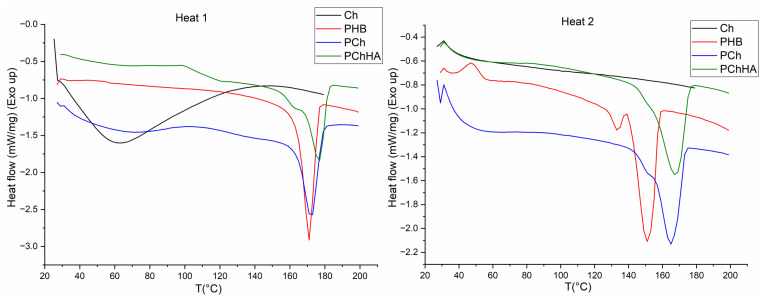
DSC curves for Ch, PHB, PCh, and PChHA10 after 1 and 2 heating cycles.

The results of the DSC analysis of thermophysical properties are shown in Figure 3. For chitosan, a typical broad endothermic peak was observed during the first heating in the 50–70 °C range, associated with the release of solvent, and this peak was absent during the second heating. A similar but significantly less pronounced peak was present in the PCh and PChHA10, which indirectly confirms the presence of chitosan in their composition. For all samples containing PHB, a narrow peak at 173 °C was observed during the first heating. A characteristic feature of the second heating for PHB was the shift of this peak toward lower temperatures (down to 150 °C) and, simultaneously, the appearance of an additional peak at 130–140 °C, which is associated with the presence of low-molecular-weight PHB in the sample, formed as a result of PHB hydrolysis by acetic acid during dissolution. It should be noted that the addition of chitosan to the PCh and PChHA10 samples led to a decrease in the peak shift from 173 °C to 163 °C and its broadening, resulting in a merger with a small peak.

In Figure 3, the PHB sample exhibits an exothermic peak in the range of 40–60 °C, which corresponds to the cold crystallization temperature (T_cc) [29].

When creating a composite from three components, the degree of crystallinity of PHB was also calculated. For pure PHB, it was 59.4%; for PHB in the PCh composite, it was 51.6%; and for PHB in the PChHA three-component composite, it was 52.2%.

### 3.3. Surface Topography Using AFM

**Figure 4:**
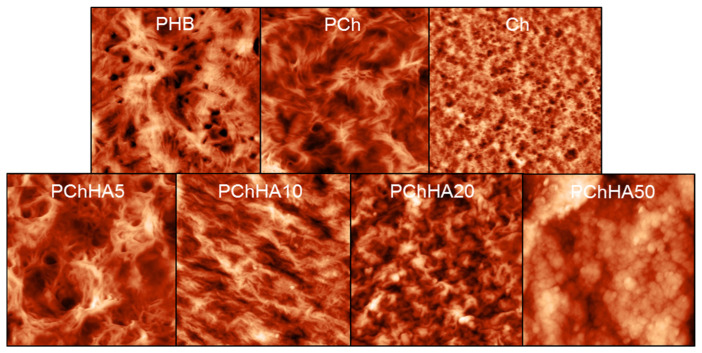
AFM images of the surface of compositions. The scanned area sizes were 20 μm × 20 μm.

The surface morphology of the samples (with different amounts of added hydroxyapatite) was examined using AFM. The results are shown in Figure 4. The root mean square (RMS) roughness was calculated and interpreted as a quantitative parameter of the surface heterogeneity of the samples; the results are presented in Table 1. Initially, before the addition of HA, the chitosan/PHB blend was studied in comparison with its individual components. Chitosan altered the structure of PHB, reducing the number of pores on the surface, while the blend formed more developed fibrous regions. Chitosan was not visualized in the microphotographs. However, its addition increased surface roughness from 50 nm to 102 nm for PHB and PCh, respectively.

The addition of 5% HA did not visually modify the topography of the composite surface. However, a further increase in its content led to a “smoothing” of the surface relief. The RMS surface roughness initially increased to 154 nm (PChHA5) and then began to decrease to 123 and 77 nm for composites containing 10% and 20% HA, respectively. Surface peaks and valleys for the PChHA10, PChHA20, and PChHA50 samples are less pronounced than for PChHA5 and PHB. A few globular HA particles and aggregates are visible on the surfaces of the PChHA10 and PChHA20 samples. As for PChHA50, it is evident that the entire surface of the sample is covered with spherical structures, which are likely hydroxyapatite and significantly alter the surface microtopography compared to the control samples. In this case, the surface roughness increased precisely due to the presence of hydroxyapatite on the surface.

### 3.4. Investigation of the Hydrophobic–Hydrophilic Properties of Scaffolds and Films

To evaluate the hydrophobic–hydrophilic properties of the composite surfaces, the water contact angles were measured both on films (model system) and on wet gels, in order to approximate the experimental conditions to those of the body’s internal environment. According to the data in Table 1, for PHB (84.6°) and PCh (74.7°) macroscopic films, it was shown that the insertion of hydrophilic chitosan into the hydrophobic PHB matrix increased the wettability of the dry surface. In addition, a gradual slight decrease in the contact angle from 83.9 to 76.3° occurred on the films as the HA content in the composition increased from 5 to 50 wt.%. The addition of 5% HA did not affect the contact angle, but as the HA content was further increased (from 10% to 50%), a gradual but steady decrease in the contact angle was observed (down to 76.3° for PChHA50).

For wet scaffolds (gels), the contact angle was significantly lower than that of the corresponding dry films in all cases. The results obtained for gels (38–62°) fell within the range from moderately hydrophilic to hydrophilic. The addition of chitosan to PHB (62.1°) made the surface more hydrophilic (51.7° for PCh). It was assumed that adding hydroxyapatite to PCh gels would gradually reduce the contact angle, with the effect increasing as the HA content in the material increased, similar to the results obtained for films. However, this was not observed in practice.

### 3.5. Water Absorption Capacity

**Figure 5:**
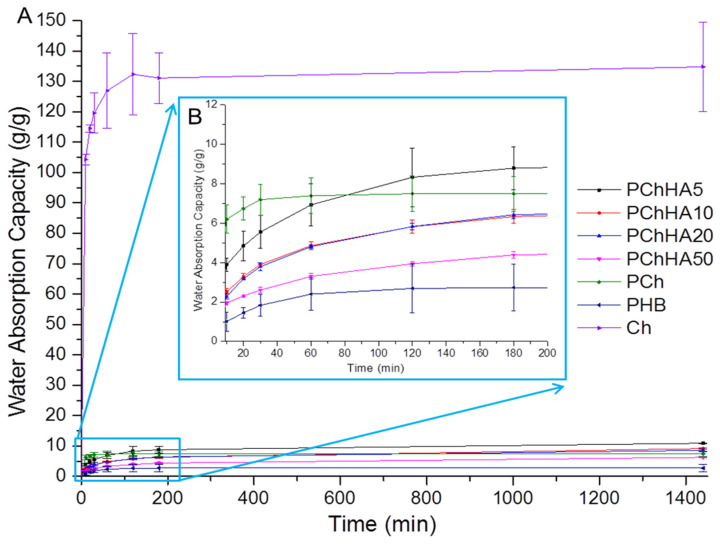
Time-dependent changes in the WAC of the scaffolds: (**A**) Equilibrium swelling; (**B**) First stage swelling for PHB-based samples.

The WAC indicates how much water a dry scaffold can absorb and retain. The study also examined equilibrium water absorption, which indicates the time interval after which the swelling process ceased. Figure 5 presents the results of the calculation of the kinetics of moisture absorption until equilibrium is reached.

Figure 5A shows that by the end of the experiment (1440 min), all samples had reached the equilibrium swelling point. For chitosan, this time was approximately 120 min; for PHB—60 min; and for PCh, approximately, 30 min. All samples containing hydroxyapatite exhibited slower swelling kinetics, which exceeded 180 min. For Ch, the water absorption was about 134 g/g, for PHB about 2.7 g/g. The addition of Ch to PHB resulted in an increase in moisture absorption to 7.5 g/g. For composites, the WAC decreased from 10.9 (for PChHA5) to 6.3 (for PChHA50). As shown in Figure 5B, after an initial increase in water absorption upon the addition of 5% HA in PCh, a steady decrease in the scaffold’s ability to absorb water was observed as the amount of hydroxyapatite in the composition increased.

### 3.6. Porosity and Density

As shown in Table 2, all samples exhibited high porosity (over 93%). The highest values were observed for the PHB sample and the PHB-chitosan sample, while the addition of hydroxyapatite in any amount slightly reduced the porosity.

The material’s density depended on its composition: the PHB sample was the densest (0.2596 g/cm^3^). The least dense of the composites was the PChHA10 (0.0601 g/cm^3^).

### 3.7. Rheological Behavior and Young’s Modulus of Scaffolds

**Figure 6:**
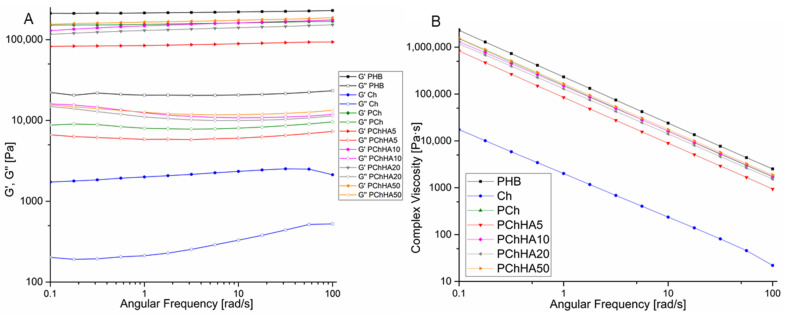
Rheological properties of scaffolds as a function of angular frequency: (**A**) Storage (G’) and loss (G”) moduli; (**B**) Complex viscosity.

The rheological properties of scaffolds based on Ch, PHB, and HA were studied using rotation rheometry. The results are shown in Figure 6 and Table 3. During the study, the viscoelastic parameters of the materials in a wet state were investigated: the values of the storage and loss moduli were determined as a function of angular frequency.

Figure 6A shows that the storage modulus was always higher than the loss modulus for all the samples. G’ and G” for chitosan are significantly lower than these parameters for the other samples. The dependence of the elastic properties of the materials on the presence of hydroxyapatite was nonlinear compared to the PCh sample. Initially, the addition of 5% HA led to a decrease in elastic properties (G’), but as its content increased, the value of G’ also increased, ultimately becoming nearly equal for PCh and PChHA50. No slope was observed for any of the curves: both the loss modulus and the storage modulus for each of the samples (except for chitosan at high frequencies) remained unchanged with increasing angular frequency. The contribution of both the viscous and elastic components to the properties of scaffolds can be quantitatively assessed by analyzing the damping factor (tan δ), the results presented in Table 3. The PHB and Ch both had tan δ approached 0.1 at an angular frequency of 10 rad/s. The damping factor for PChHA10 (0.064) was the lowest among all composites.

The results shown in Figure 6B demonstrated a significant decrease in complex viscosity for all scaffolds as the angular frequency increased from 0.1 to 100 rad/s. Initially, the viscosity of all samples, except chitosan, was in the range of 1 MPa·s, indicating high structural stability of the scaffolds and sufficient mechanical stiffness.

In addition, the values of the compressive Young’s modulus were analyzed; the results are shown in Table 3. Young’s modulus represents stiffness as resistance to deformation. The PHB scaffolds were the stiffest; the addition of chitosan to them reduced the Young’s modulus by more than half, to 12.3 kPa, while the incorporation of hydroxyapatite changed the stiffness of the samples only slightly.

### 3.8. In Vitro Biodegradation in PBS and in an Enzyme Solution

**Figure 7:**
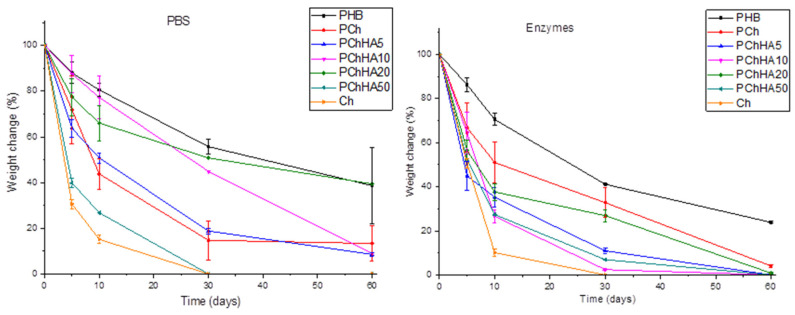
Study of the degradation process of scaffolds in PBS and enzyme solution for 60 days.

The effect of scaffold composition was evaluated during *in vitro* biodegradation in the presence of lipase and lysozyme enzymes and in phosphate-buffered saline; the results are shown in Figure 7. This is necessary to assess the effect of enzymes on the stability of the scaffolds over time under conditions that mimic the internal environment of the body.

Hydrolytic degradation in PBS simulated the degradation kinetics in the absence of chemical agents. As shown in Figure 7, PHB degraded quite slowly, losing about 10% of its mass by day 5 of the experiment and about 60% by day 60. The chitosan scaffolds swelled during the first day and lost their structure, completely degrading within 30 days. The PCh sample lost more than half of its mass between days 5 and 10 of the experiment. Enzymatic degradation was characterized by a more pronounced decrease in mass for all samples in the first days of the experiment, compared to hydrolytic degradation. The total weight loss of the PHB and Ch scaffolds after 60 days was also significantly higher, which is attributed to the presence of lipase, which breaks the ester bonds of PHB, and lysozyme, which acts on the β-(1→4)-glycosidic bonds in chitosan. The addition of hydroxyapatite had a nonlinear effect on the degradation rate of the scaffolds in both PBS and the enzyme solution. However, the degradation of PCh in the enzyme solution proceeded more slowly than in PBS and more slowly than when any amount of hydroxyapatite was added to the mixture, up to 30 days into the experiment. In PBS, among all composites, PChHA10 and PChHA20 degraded the slowest; in the enzyme solution, only PChHA20 approached the degradation rate of PCh, while all other composites lost more than 80% of their mass by day 30.

### 3.9. Cytocompatibility

**Figure 8:**
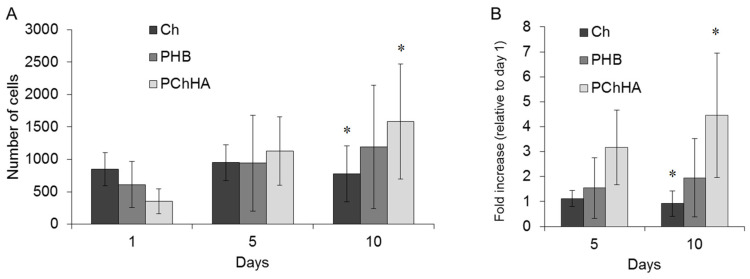
Cytocompatibility study: (**A**) Number of attached cells; (**B**) Cell proliferation dynamics on the test samples by day 10 of cultivation. *—statistically significant differences between groups (*p* < 0.05).

A biocompatibility study was conducted on the most promising PChHA10 composite sample using MSC line; the results were evaluated after 1, 5, and 10 days of incubation. The data are shown in Figure 8.

The number of cells on PHB, chitosan, and the selected material containing 10% HA was assessed, and the fold increase in cell count was calculated on days 5 and 10 of the experiment. It was shown that Ch virtually did not support proliferation. By day 5, the number of attached viable cells had increased slightly (1.1-fold), and by day 10, it had decreased compared to day 1. PHB showed stable proliferation growth: a 1.95-fold increase in cell count by day 10. PChHA demonstrated the best proliferative activity: a 4.45-fold increase in cells after 10 days. This experiment showed that it was precisely the addition of HA that improved the cytocompatibility of the polymer matrix due to the synergistic effect of its components, manifested in the enhancement of cell-friendly surface properties.

## 4. Discussion

The formation of composites based on PHB, Ch, and HA was evaluated using different methods. FTIR spectroscopy results showed that in the PCh spectrum, the characteristic peaks of both polymers overlapped. The shift of the PHB carbonyl peak to the low-frequency region and the decrease in the intensity of the chitosan amide region may indicate the formation of hydrogen bonds between the polymers [30]. The addition of HA (PChHA) had virtually no effect on the overall spectral pattern, indicating little influence of the inorganic filler on the polymer composite structure.

During a second heating DSC analysis, a typical narrow melting peak at 173 °C was shifted to 150 °C, with the appearance of an additional peak at 130–140 °C. This behavior is associated with the hydrolysis of polymer chains by acetic acid during dissolution and heating, resulting in the presence of low-molecular-weight PHB in the mixture [31]. The addition of chitosan and HA (PCh, PChHA) shifted the PHB peak to 163 °C and broadened it, which is consistent with literature reports [32,33,34]. This effect is attributed to rigid chitosan chains restricted the molecular mobility of PHB, thereby hindering the formation of large crystals. The endothermic peak at 163 °C thus confirms interphase interactions between the biopolymers, leading to partial amorphization of the PHB structure in the presence of chitosan and hydroxyapatite. Since the melting point of hydroxyapatite typically exceeds 1100 °C [35], its incorporation into the composite produced no additional peaks within the scanned temperature range. The crystallinity of the PChHA was 52.2%, which differed only slightly from the crystallinity of the sample without HA (PCh)—51.6%. These data are consistent with those in the literature, which also state that HA has a negligible effect on the degree of crystallinity of PHB [36]. At the same time, the degree of crystallinity of pure PHB was significantly higher, indicating that it was chitosan, rather than HA, that had a significant effect on the crystallization of PHB within the composite.

A series of composite scaffolds with a fixed polymer ratio (4:1 by weight for PHB and Ch, respectively) and varying amounts of HA (5%, 10%, 20%, and 50%), were prepared. HA concentrations below 5% did not significantly affect composite properties, whereas HA content exceeding 50% increased brittleness, induced toxicity, and promoted the formation of large, non-resorbable aggregates. AFM analysis revealed that both the presence of Ch and varying HA content alter the morphology of the PHB-based composite. Increasing HA content from 5% to 20% smoothed the surface topography, acting as a physical filler. At 50% HA, the surface was uniformly covered with spherical structures, likely representing hydroxyapatite aggregates. Sample density increased with HA addition (0.06–0.067 g/cm^3^) compared to PCh (0.056 g/cm^3^), which also confirms the role of HA as a physical filler.

The surface contact angle is most often determined using films. This approach enables assessment of each film component contribution to surface hydrophobicity and hydrophilicity; however, it does not account for the morphology and surface structure of the target material. Measuring hydrophobic properties on both films and scaffolds is important because it addresses different physical states of the composite material. In this study, the target materials are three-dimensional scaffolds; therefore, we evaluated and compared contact angle values both on films and on the surface of scaffolds (simulating a humid microenvironment approximating real conditions). This comparison was feasible because the scaffold surface before drying (gels) was dense and non-porous. Consequently, a water droplet did not seep inside the scaffold but instead slowly spread across its surface. The addition of 5% HA did not significantly affect the contact angle of the films. However, further increases in HA content led to a gradual decrease in the contact angle. This suggests that at higher concentrations, HA begins to dominate the surface composition, consistent with AFM observations. The increased hydrophilicity of the composites compared to pure PHB can be attributed to the presence of polar hydroxyl and phosphate groups in the hydroxyapatite structure. In contrast to the chitosan sample, all films containing HA exhibited a higher contact angle. This may result from HA particles on a smooth surface creating additional micro-roughness, which enhances the natural hydrophobicity of PHB, the primary component of the composite. Overall, the contact angle values for the films (>65°) indicate moderately hydrophobic surfaces.

The contact angle measured on gels (38–62°) was significantly lower than that on dry films (66.8–84.5°), consequently, all three-dimensional materials can be classified as hydrophilic. This difference likely arises from swelling and hydration of the composite material. The gel surface was soft, dynamic, and moisture-rich substantially increasing its wettability. In practice, such properties are more favorable for biological interactions (including cell adhesion and wetting by tissue fluid). For gels, the effect of chitosan adding was more pronounced. For PHB (62.1°) and PCh (51.7°) it was suggested that, in the wet state, hydrophilic groups of chitosan become active, migrate to the interface with water, and significantly hydrated it.

The inclusion of HA did not produce a linear change in the hydrophobic–hydrophilic properties. An optimal HA concentration (10%) was identified at which material hydrophilicity is maximized. PChHA10 exhibited a minimum contact angle of approximately 38.9°. This likely results from an optimal balance between HA-induced hydration and the surface properties of the polymer matrix (PHB/Ch), including roughness, porosity, and water absorption. Migration of hydrophilic components to the gel surface may also contribute to this effect. Previous studies have reported that contact angles of approximately 40–60° promote good cell adhesion [37,38]. Therefore, the PChHA10 sample can be considered a promising for cell adhesion.

Materials designed for biomedical applications need to be able to absorb moisture in a controlled manner, as they are typically used in the body’s humid environment. Experiments investigating water absorption have shown that pure chitosan absorbs the maximum amount of liquid (approximately 130 g/g), but the scaffold structure degrades rapidly. It is known that excessive moisture absorption by chitosan is often a drawback in the development of biomaterials based on it and limits its use in its unmodified form [39]. PHB interacted with water to a negligible extent and exhibited the lowest moisture absorption (approximately 2.7 g/g), likely due to the physical penetration of water into the material’s pores. Blending chitosan and hydrophobic PHB resulted in the transformation of highly swellable Ch into an inert, slowly and weakly swellable matrix (PCh). In such a composite, the swelling process is limited primarily by the slow diffusion of water through the dense hydrophobic PHB phase. The addition of HA made the scaffolds denser, which hindered and slowed the diffusion of water into the sample. The addition of 5% HA slightly increased the WAC, but further increases in HA content monotonically decreased absorption. This effect requires further analysis, which we will explore in future work. But we can assume that HA slowed diffusion, making the material’s structure denser. Presumably, hydroxyapatite acted as a structural modifier that began to fill the scaffold’s volume and hindered initial swelling (thereby reducing its rate), creating an additional physical barrier to water diffusion. Hydroxyapatite, as an inert filler, physically filled the scaffold material, as evidenced by the increase in density, which we had previously hypothesized.

Rheological test data show that the samples are viscoelastic materials. This means that no phase transition occurred, since the moduli did not cross at any of the angular frequencies studied. It is important to note the absence of frequency dependence in the moduli, indicating that the structure of the samples is stable under different loads. The damping factor for all composites is below 0.07 (except for Ch), classifying them as “strong” gels in which elasticity predominates over viscosity. It is known that the smaller this value is, the stronger the elastic properties of the material. The PHB and Ch samples can be classified as more “weak,” since tan δ approached 0.1. Authors [29] reported that a low ratio of the loss modulus to the storage modulus allows a strong gel (0.01 and below) to be distinguished from a weak one (around 0.1). The damping factor for PChHA10 (0.064) was the lowest among all composites, indicating high elasticity and the ability to withstand cyclic loading. An investigation of complex viscosity showed that all samples containing PHB exhibited pseudoplastic thinning under shear typical of most polymers [40], and high structural stability (~1 MPa·s). A significant decrease in complex viscosity under shear may be useful in the development of electrospun or injection-molded materials. Stiffness (Young’s modulus in compression) decreased both upon the addition of chitosan and in the presence of hydroxyapatite. The authors of [41] also reported a deterioration in the mechanical properties of PHB-based composites as the hydroxyapatite content increased from 5% to 20%, which is consistent with our results. They attributed this to poor atomic bonding between the PHB and HA materials. The large difference in melting points between the two materials prevented them from interacting at the molecular level during mixing at low temperatures. Furthermore, due to their high surface energy, HA nanoparticles tended to agglomerate very rapidly at higher concentrations, which also impaired their interaction with PHB and contributed to a decrease in the Young’s modulus of the composites. However, at the highest HA content (50%), this tendency was not observed, which may be due to the fact that the mechanical properties of hydroxyapatite itself begin to exert a greater influence on the properties of the blend.

Studying the rate of biodegradation under conditions that mimic the body’s internal environment is extremely important for biomaterials that may eventually be used in medicine. Biopolymers have different rates of degradation; for example, PHB typically degrades in the body over an extremely long period (>1 year), while its modification, including blending with other polymers and low-molecular-weight components, allows for controlled acceleration of biodegradation [42]. In our study, degradation processes were investigated in PBS as a model medium, and in PBS supplemented with pancreatic lipase and lysozyme, the primary enzymes capable of degrading PHB and chitosan in a living organism. In a previous study, we demonstrated that the addition of chitosan to PHB can controllably influence the rate of PHB degradation [27]. Degradation of all samples in the enzyme solution occurred significantly faster. All composites except PChHA20 lost more than 80% of their mass by day 30. This indicated that neither too small nor too large amounts of hydroxyapatite contributed to the stabilization of the material’s internal structure, despite the fact that the samples with PChHA5, PChHA10, and PChHA20 had very similar rheological properties. At the same time, the most stable composites were the PChHA10 and PChHA20 samples, while PChHA50 exhibited a high degradation rate in both cases, likely due to an excess of HA leading to inhomogeneities in the material. This indicates a certain optimal hydroxyapatite content (10–20%) at which the sample structure becomes most stable and its degradation proceeds more slowly and uniformly.

It is important to note that our composites are based on biodegradable polymers that are completely broken down by the body’s metabolic pathways without causing any toxic effects. Chitosan and its degradation products are generally considered safe, exhibiting low toxicity to terrestrial and aquatic organisms [43]. Enzymatic hydrolysis of chitosan leads to the formation of chitooligosaccharides, and upon complete degradation, the main product is the monomer D-glucosamine (with N-acetyl-D-glucosamine formed to a lesser extent). This monomer is a natural precursor for the synthesis of glycosaminoglycans. PHB is broken down into monomers (D-3-hydroxybutyrate), which are then oxidized in tissues to acetoacetate and ultimately excreted from the body as carbon dioxide and water [17]. The dissolution of HA leads to the release of calcium ions (Ca^2+^) and phosphate ions (PO_4_^3−^) into the environment. Calcium and phosphate ions are natural components of extracellular fluid and bone tissue. The release of these ions may stimulate the formation of new bone tissue. However, the dissolution rate of pure HA in a neutral medium (pH 7.4) is extremely low. Some research demonstrates that, despite their biocompatibility, HA nanoparticles may exhibit cytotoxic effects in certain cases [44]. The toxicity of HA nanoparticles is determined by their size and aggregation capacity [45]. High local concentrations of aggregated HA nanoparticles enhance the production of reactive oxygen species and increase cytotoxicity. However, in the polymer system (chitosan/PHB), local acidification of the medium may occur due to polymer hydrolysis processes, which could accelerate the decomposition of hydroxyapatite into bioactive, non-toxic components (calcium and phosphate ions) and prevent aggregate formation. Therefore, a limiting factor of this study and an important direction for further work will be the investigation of hydroxyapatite degradation in the composite, control of nanoparticle aggregation, and additional monitoring of the release of calcium and phosphate ions.

Based on all the above-described experiments, a nonlinear relationship was observed between the investigated properties of the composites and the concentration of hydroxyapatite in the composition. Neither the highest (50%) nor the lowest (5%) content of this component led to an improvement in the physicochemical and structural properties of the composite. However, the PChHA10 sample can be considered the most balanced in terms of its overall properties, as it demonstrated a compromise between hydrophilicity, moisture absorption, mechanical strength, and stability against degradation. In contrast to PChHA5 (less hydrophilic, degrading faster) and PChHA20/50 (more hydrophobic and mechanically weaker), the composite with 10% HA exhibited a synergistic effect of all components. Therefore, this sample was further investigated for biocompatibility. It was shown that the presence of hydroxyapatite specifically promoted MSC proliferative activity of the composite surface, compared to control samples.

Overall, our results indicate that the PChHA10 composite is suitable for further *in vitro* studies (cultivation of osteoblasts and chondrocytes) and *in vivo* studies (implantation in models of bone and cartilage defects), as it represents the most balanced combination of physicochemical, mechanical, and biological properties.

## 5. Conclusions

In this study, scaffolds composed of PHB and chitosan polymers and reinforced with hydroxyapatite were prepared and investigated. FTIR and DSC analyses showed that hydroxyapatite did not significantly affect the structural and thermophysical properties of the materials. However, the addition of HA altered the surface morphology of the samples and affected the roughness values. Most of the investigated parameters, including moisture absorption, biodegradation rate, Young’s modulus, porosity, and hydrophobic/hydrophilic properties, changed nonlinearly as the hydroxyapatite content increased from 5% to 50%. It was found that at a 10% HA content, the composition exhibited the best stability and optimal viscoelastic properties, a hydrophilic surface with a contact angle of approximately 39°, an RMS roughness of approximately 123 nm, and acceptable rates of degradation and swelling. The presence of hydroxyapatite ensured good proliferative activity of the composite toward MSCs, as evidenced by a 4.45-fold increase in cell numbers after 10 days of cultivation compared to day 1. Overall, PHB/Ch/HA scaffolds are promising biomaterials and could serve as a basis for the development of new products for tissue engineering and regenerative medicine based on biopolymers; however, further research is required to realize this potential. The results obtained in this study contribute to our understanding of the behavior of multicomponent systems composed of polymers and inorganic materials, which is important for further research in this field.

## Figures and Tables

**Table 1:** Analysis of RMS roughness and contact angle for films and scaffolds.

Sample	RMS Roughness, nm	Contact Angle, °	
		Films	Scaffolds
PHB	50.1 ± 0.2	84.6 ± 2.2 ^a^	62.1 ± 4.3 ^a^
PCh	102.1 ± 15.8	74.7 ± 8 ^c^	51.7 ± 6.5 ^ab^
PChHA5	154.2 ± 27.7	83.9 ± 8.8 ^a^	53.9 ± 8.5 ^ab^
PChHA10	123.8 ± 3.3	81.3 ± 5.2 ^ab^	38.9 ± 6.5 ^c^
PChHA20	77.2 ± 2.4	80.1 ± 5.8 ^ab^	41.4 ± 3.7 ^c^
PChHA50	111.4 ± 27.4	76.3 ± 5.2 ^bc^	46.8 ± 13.1 ^bc^

^abc^ Different superscript letters within the same column indicate statistically significant differences between groups (*p* < 0.05).

**Table 2:** The results of porosity and density calculations for the scaffolds.

Sample	Porosity, %	Density, g/cm^3^
PHB	95.97 ± 3.55	0.2596 ± 0.44
PCh	96.30 ± 0.89	0.0563 ± 0.01
PChHA5	93.26 ± 3.17	0.0674 ± 0.03
PChHA10	94.62 ± 1.14	0.0601 ± 0.01
PChHA20	93.36 ± 3.46	0.0664 ± 0.03
PChHA50	93.26 ± 2.07	0.0674 ± 0.02
Ch	N/a	N/a

**Table 3:** Young’s modulus and damping factor (tan δ) of PHB-based composites reinforced with hydroxyapatite.

Sample	Young’s Modulus, kPa	Damping Factor (tan δ)
PHB	27.171 ± 8.135 ^a^	0.092 ± 0.014 ^a^
Ch	1.632 ± 0.783 ^b^	0.179 ± 0.033 ^b^
PCh	12.359 ± 3.213 ^c^	0.067 ± 0.015 ^c^
PChHA5	10.834 ± 0.789 ^c^	0.068 ± 0.003 ^c^
PChHA10	10.063 ± 2.337 ^c^	0.064 ± 0.007 ^c^
PChHA20	9.158 ± 3.830 ^c^	0.070 ± 0.011 ^c^
PChHA50	11.176 ± 1.537 ^c^	0.068 ± 0.005 ^c^

^abc^ Different superscript letters indicate statistically significant differences between groups (*p* < 0.05).

## Data Availability

The raw data supporting the conclusions of this article will be made available by the authors on request.

## Abbreviations

The following abbreviations are used in this manuscript:

AFM	Atomic force microscopy
DSC	Differential scanning calorimetry
FTIR	Fourier transform infrared spectroscopy
WAC	Water absorption capacity
RMS	Root mean square
PHB	Polyhydroxybutyrate
HA	Hydroxyapatite
MSC	Mesenchymal stem cells
